# Supporting Adolescents and Young Adults through Digitally Mediated Type 1 Diabetes Transition Care: A Qualitative Descriptive Study

**DOI:** 10.1155/2024/3721768

**Published:** 2024-07-15

**Authors:** Noor El-Dassouki, Madison Taylor, Kaylen J. Pfisterer, Ashish Saragadam, Meranda Nakhla, Marley Greenberg, Alanna Landry, Geetha Mukerji, Elise Mok, Anne-Sophie Brazeau, Jessica C. Kichler, Joseph A. Cafazzo, Rayzel Shulman

**Affiliations:** ^1^ Centre for Digital Therapeutics University Health Network, 190 Elizabeth Street Toronto, M5G 2C4, Toronto, ON, Canada; ^2^ Department of Systems Design Engineering University of Waterloo, 200 University Avenue W, Waterloo N2L 3G1, ON, Canada; ^3^ School of Public Health Sciences University of Waterloo, 200 University Ave W, Waterloo N2L 3G1, ON, Canada; ^4^ Division of Endocrinology Montreal Children's Hospital McGill University Health Centre, 1001 Boulevard Decarie, Montreal, QC H4A 3J1, Canada; ^5^ Research Institute of the McGill University Health Centre, 2155 rue Guy, Montreal H3H 2R9, QC, Canada; ^6^ Diabetes Action Canada Toronto General Hospital, 200 Elizabeth Street, Toronto M5G 2C4, ON, Canada; ^7^ Markham Stouffville Hospital Oak Valley Health, 381 Church Street, Markham L3P 7P3, ON, Canada; ^8^ Women's College Hospital, 76 Grenville Street, Toronto M5S 1B2, ON, Canada; ^9^ Centre for Outcomes Research and Evaluation Research Institute of the McGill University Health Centre, 5252 de Maisonneuve Boulevard W., Montreal H4A 3S9, QC, Canada; ^10^ School of Human Nutrition McGill University, 21111 Lakeshore Road, Ste. Anne de Bellevue H9X 3V9, QC, Canada; ^11^ Department of Psychology University of Windsor, 401 Sunset Avenue, Windsor N9B 3P4, ON, Canada; ^12^ Institute of Health Policy Management and Evaluation Dalla Lana School of Public Health University of Toronto, 155 College Street, Toronto M5T 3M7, ON, Canada; ^13^ Institute of Biomedical Engineering University of Toronto, 164 College Street, Toronto, ON M5S 3E2, Canada; ^14^ Healthcare Human Factors University of Toronto, 190 Elizabeth Street Toronto, Toronto M5G 2C4, Canada; ^15^ SickKids Research Institute, 686 Bay Street, Toronto M5G 0A4, ON, Canada; ^16^ Institute for Clinical Evaluative Sciences, 2075 Bayview Avenue, Toronto M4N 3M5, ON, Canada; ^17^ Division of Endocrinology The Hospital for Sick Children, 555 University Avenue, Toronto M5G 1X8, ON, Canada

## Abstract

**Objective:**

The time during which adolescents and young adults (AYAs) living with Type 1 Diabetes (T1D) transition from pediatric to adult care is associated with blood sugar levels outside of target ranges, care gaps, and an increased risk of acute diabetes complications. The aim of this study was to understand (1) the perspectives of AYAs and providers about the strengths, challenges, and opportunities of transition care and (2) the role of digital technologies in supporting the transition to adult care. *Research Design and Methods*. We conducted a qualitative descriptive study that involved 43 semistructured interviews in French or English with AYA living with T1D (aged 16–25; *n* = 22) and pediatric or adult diabetes health care providers (HCPs) (*n* = 21).

**Results:**

We identified three themes. First, transition care is not standardized and varies widely, and there is a lack of awareness of transition guidelines. Second, virtual care can simultaneously hinder and help relationship-building between providers and AYA. Third, AYAs value a holistic approach to care; both HCPs and AYA highlighted the opportunity to better support overall mental wellbeing.

**Conclusions:**

The design of digital technologies to support T1D transition care should consider methods for standardizing holistic care delivery and integrating hybrid diabetes care visits to support access to transition care. These findings can inform future transition intervention development that leverages existing transition guidelines, targets holistic care model integration, and considers quantitative diabetes metrics in conjunction with broader life experiences of AYA when providing transition care.

## 1. Introduction

The transition from pediatric to adult Type 1 Diabetes (T1D) care occurs around age 18 for adolescents and young adults (AYAs) in Ontario and Quebec, Canada. It is fraught with complexity from two main factors. First, the changing care environment is associated with a reduction in support and resources for AYA, coupled with increased responsibility for AYAs' T1D care [1, 2]. Second, the transfer to adult care coincides with emerging adulthood, a developmental life stage wherein AYAs have competing life priorities from postsecondary education, work and career opportunities, changes in living arrangements, and peer relationships [3]. This results in many AYAs not feeling adequately prepared for the pediatric to adult care transition [4], and deterioration of glycemic management [2], decreased clinic attendance [5], and increased risk of diabetes-related hospitalizations [6] often associated with the transition.

Currently, no single transition program is effective. Transition interventions targeting patients, clinic staff, and services show mixed results in improving outcomes, including glycemic management and clinic attendance [7]. Existing interventions involve technology-facilitated education (e.g., web-based modules) and dedicated clinic resources (e.g., young adult clinic and transition coordinators) [7]. Given the lack of transition interventions' efficacy evidence, evaluation is needed for new innovations designed and tested for feasibility, scalability, adoptability, and spreadability to support the wellbeing of AYAs across the transition process. Some generic transition guidelines exist, such as the Got Transition toolkit [8], as well as specific system-level recommendations for T1D transition (e.g., American Diabetes Association's transition care statement) [9]. These guidelines provide a structured approach to transition care. Both the Got Transition [10] resources and the Ontario Pediatric Diabetes Network have a transition implementation guide to facilitate implementation [11]. However, in practice, uptake of implementation guidelines has been poor, owing to barriers from lack of standardization of transition care protocols and staff training [12, 13].

Additionally, the COVID-19 pandemic caused a shift towards T1D care delivery through virtual and hybrid (which combine virtual and in-person care) modalities [14]. It is difficult to measure the effect of diabetes virtual care delivery [15]; little is known about patients' and providers' experience with it. However, virtual care may help overcome some barriers to in-person diabetes care [15]. We refer to virtual care as any digital technology-mediated healthcare interaction (e.g., person-to-person, app-to-person, and person-to-app) via a digital health tool. Digital health tools provide technological infrastructure to deliver virtual care (e.g., mobile/web applications to deliver education, computer games, text messaging, reminders, and support during transition). Digital health tools can enhance quality of life [16], patient-centered care [17], self-efficacy or self-management [18], availability, and affordability of services while optimizing patients' and health care providers' time [17], and timely access to care [16, 17]. However, it is unknown if existing digital tools meet AYA or health care providers' (HCPs) needs [19]. The aim of our study is to explore perspectives of AYAs living with T1D and T1D HCPs about (1) transition process strengths, challenges, and opportunities and (2) how digital health can support transition.

## 2. Methods

### 2.1. Study Design

This qualitative descriptive study was conducted between Feb 2021 and April 2022 in Ontario and Quebec, Canada. The research team (Noor El-Dassouki, Madison Taylor, and Ashish Saragadam) collected and analyzed data at the University Health Network (UHN) in Toronto. This study was approved by the following Research Ethics Boards: UHN (REB 19-6247), SickKids (REB 1000065721), Oak Valley Health (REB 106-2002), Women's College Hospital (REB 2020-0032-E), and McGill University Health Centre (REB 2020-6241).

#### 2.1.1. Setting and Participants

We recruited a convenience sample of AYA living with T1D as well as pediatric and adult diabetes HCPs from T1D clinics (SickKids Hospital, Oak Valley Health, Women's College Hospital, and McGill University Health Centre (Montreal Children's Hospital and Royal Victoria Hospital)), and study advertisements shared by the BETTER registry, ConnecT1D Canada, the Ontario Pediatric Diabetes Network, and Diabetes Action Canada. We aimed to recruit an equal number of participants from each province to reduce proportional bias and capture a broad range of experiences. Written informed consent was obtained from all participants.

#### 2.1.2. Positionality of the Research Team

We outlined our team's positionality [20, 21] in *Supplementary file 1*. Positionality encapsulates how researchers' lenses (i.e., identities, values, experiences, and social positionings) shape interactions with and influence data collection, analysis, and interpretation [20, 21]— a crucial element of ensuring rigour in our qualitative analysis.

#### 2.1.3. Data Collection

*(1) Interviews*. We conducted 1 hr virtual semistructured interviews in English or French via Microsoft Teams or phone. Interview guides were informed by current state service mapping interviews conducted with staff at study sites to map out existing T1D transition care services provided to AYA and refined through input from study investigators. The interview guide was pilot-tested with clinician and patient partner stakeholders (*Supplementary file 2*). Interviews were audio recorded and transcribed. Demographic data were collected in an electronic survey distributed through Typeform [22] (*Supplementary file 3*). In alignment with qualitative principles of theoretical data saturation, our team conducted thoughtful iterative analyses throughout the data collection period to (re)assess the need for additional interviews as part of the process of saturation that enabled us to achieve conceptual depth [23].

#### 2.1.4. Analysis

*(1) Codebook Development*. Authors Noor El-Dassouki and Madison Taylor reviewed four transcripts to formulate an initial codebook for systematically coding transcript themes [20]. The codebook was independently applied to two additional transcripts by Noor El-Dassouki, Madison Taylor, and Ashish Saragadam and iteratively refined to remove ambiguity and overlap. The finalized codebook was used for formal analysis.

*(2) Analysis of Transcripts*. Noor El-Dassouki, Madison Taylor, and Ashish Saragadam conducted a thematic content analysis of the interview data [20] using NVivo software (Version 12) [24]. Each coder independently coded transcripts. Themes were informed by interview guide topics and inductive reasoning. Demographic characteristics of study participants were summarized with descriptive statistics.

Guided by quality qualitative research criteria [25], we engaged in reflexivity throughout the study, detailed our positionality, captured multivocal perspectives of those with lived experience (patients and clinicians) in a large sample, and grounded analysis within a theoretical framework (i.e., the socioecological model [26]). This tiered perspective robustly identifies gaps to facilitate understanding health experiences and behaviors influenced by a combination of factors (e.g., individual characteristics, organizations, and systems) [26]. We applied a strengths-based approach framing research within the positive context of the strengths that individuals and organizations already possess [27] to translate opportunities to concrete actionable steps.

## 3. Results

Forty three (28%) AYAs living with T1D and HCPs participated in semistructured interviews (AYA: 76 interested and 22 interviewed; HCPs: 57 interested and 21 interviewed). Nineteen (86%) AYA identified as White, with 11 (50%) 16–17 years old and 11 (50%) 18–25 years old. Seventeen (77%) of the sample used insulin pumps, which has been used as a proxy for financial privilege in Ontario [28]. *Supplementary tables 1* and *2* provide full sample demographics. Thirteen of AYAs were pretransfer to adult care and the remaining 9 were posttransfer. HCP roles were endocrinologists (*n* = 5, 24%), nurses (*n* = 5, 24%), dietitians (*n* = 5, 24%), and other (social workers, pharmacists, and general pediatricians; *n* = 6, 29%). HCP experience ranged from 1 to ≥ 11years.

We identified three themes: (1) transition care is not standardized and varies widely, (2) virtual care can hinder or help relationship building between AYAs and HCPs, and (3) AYAs value a holistic T1D approach over quantitative metrics of glycemic management. Each theme is explored below, framed as challenges and strengths from the perspective of AYA with T1D and HCPs.

### 3.1. Transition Care Delivery Is Not Standardized and Varies Widely

HCPs described that lack of clinic resources (i.e., not enough clinicians, and especially those with specific expertise such as mental health) limits the time and opportunity that clinicians have to provide comprehensive transition care. This leads to a wide variability of transition practices across clinics. HCPs noted that transition care provided is dependent on clinician availability; many clinics do not have standardized practices to prepare AYAs for transition. Furthermore, when asked about national and provincial guidelines and policies to support transition, many HCPs noted either a lack of familiarity with existing resources or a lack of transition-specific content.

*“We don't really have any guidelines or forms that we make here, really official to guide us in this transition.” [Pediatric HCP, translated]*

*“I think it would be so helpful to have more of a standard approach to first [adult clinic] visits and ongoing visits and information to collect and all that stuff. And if it exists, I don't really honestly know about it.” [Adult HCP]*

HCPs noted that even if they have transition practices, staffing, and space resource constraints affect the ability for clinics to implement standardized transition practices. HCPs noted AYAs have different experiences of transition preparation practices, processes, and experiences depending on the availability and capacity of individual HCPs at the clinic they attend.

*“It's a very, case-by-case and physician-by-physician approach.” [Pediatric HCP]*

*“I think we should have a systematic [approach]. Oftentimes, the main problem is that we have 500+ patients and when we see them, it's not always possible to see them every time they come.” [Pediatric HCP, translated]*

Despite the variability, HCPs noted clinician adaptability facilitates supportive transitions. During the COVID-19 pandemic, clinicians noted significant pandemic-related changes in transition care. Specifically, HCPs noted the integration of virtual care and digital health tools, including the use of social media platforms and adaptation of transition education into virtual platforms.

*“So, in the pre-COVID times, we implemented a transition readiness questionnaire, so we ask a specific set of questions with both the individual and family member or caregiver in the room so we can start talking about [task completion] before they actually move on to being fully independent. So now during COVID, we provide a series of three webinars, talking about tasks to practice between now and the [transition] time.” [Pediatric HCP]*

AYAs and HCPs valued digital tools to support transition care. Specific suggestions from participants included reminder tools, providing educational resources to guide AYAs through a transition curriculum, and helping AYAs access their power for self-advocacy and decision making around transition and healthcare.

*“I think for reminders, let's say I have an appointment coming up, maybe a reminder … a few days before [to] just get a text message or something like that with a heads up.” [Pre-Transition AYA]*

### 3.2. Virtual Care Can Hinder and Help Relationship-Building

Participants noted the challenges of building rapport with new adult diabetes providers during virtual diabetes care appointments. AYAs and HCPs reported conducting virtual visits in spaces that were not private made it difficult to communicate openly and build trust.

*“[COVID has] made a little bit of that relationship forming aspect more difficult, because now we're dealing with phone calls as check-in appointments.” [Post—Transition AYA]*

*“We've transitioned to mostly virtual care, which doesn't lend itself well to independent interviews [with AYA] – and with no guarantee that the parents [can't hear the conversation]” [Pediatric HCP]*

HCPs also noted AYA without access to technology are disadvantaged because they cannot access the benefits of virtual diabetes care.

*“Because lack of internet, lack of reliable internet, lack of money for a data plan, that's something we have struggled with, with some of our families, and so that makes it difficult… they can't even access the support that we can provide them. [Pediatric HCP]*

For those who do have access to technology and have digital proficiency, virtual care can add convenience.

*“So the endocrinologist is more flexible, more available to have a meeting like that and it's very fine with me to have it on the telephone.” [Post-Transition AYA]*

*“I guess [only having virtual appointments] is one of the nice things coming out of this pandemic… they can kind of reach me wherever I am.” [Pre-Transition AYA, moving away for school]*

Furthermore, participants shared the potential of virtual care to support a positive relationship between AYAs and their HCPs. Specifically, virtual care can improve access to care by reducing the need for travel and time lost to attend appointments, along with transportation barriers and costs. Further, virtual care can provide greater flexibility around appointment scheduling and improve access to connect with HCPs regardless of location. As noted by participants, this is especially poignant for AYA experiencing mental health challenges, where symptoms like fatigue impede capacity to attend appointments.

*“Getting out of bed, getting in a car or getting on a bus and getting to an appointment, it's a lot…for some of those patients, being able to do it from home where they're more comfortable, or being off camera, that has made things more accessible as well.” [Adult HCP]*

HCPs saw value in digital tools (e.g., mobile/web applications, computer games, text messaging, reminders, and support during transition) to personalize transition care, and provide a medium for AYAs to access care and education when they need it, without adding to HCPs workload. HCPs reported using digital tools for delivering education and transition curricula could increase their efficiency during clinics.

*“The things that would be good about apps would be automating [the transition checklist] a bit more. So if an app had … some sort of interactive module where the patient enters in their own responses that could get sent back to the provider would be really good…we have time constraints, patients have time constraints.” [Adult HCP]*

### 3.3. AYAs Value a Holistic Type 1 Diabetes Approach over Quantitative Metrics of Glycemic Management

AYAs and HCPs reported clinicians' emphasis on traditional measures of glycemic management negatively affect AYAs attitudes towards T1D self-care. Specifically, AYAs reported feeling judged by their clinicians when HCPs place a heavy emphasis on hemoglobin A1c (HbA1c) values and daily blood glucose levels. This led AYAs to feel like a “bad patient”.

*“… I would go to my appointments and they would really just sit and look at every single blood sugar that I had for like the past three months and like really kind of scrutinize me, and I would get in trouble. And so every time that I like went to the endo I kind of associated it with getting in trouble.” [Post-Transition AYA]*

HCPs and AYAs described how perceived judgements and attitudes make AYAs feel pressured, discouraged, and overwhelmed by their care already strained by managing other life stressors, roles, and responsibilities.

*“If I'm {…} feeling stressed or overwhelmed and, I can't complete everything in my life, one of the first things that I drop is my diabetes care” [Post-Transition AYA]*

In addition, the perception of feeling judged by HCPs makes it increasingly difficult for AYA to trust their HCP. These combined experiences can result in care gaps during transition, especially when building trusting relationships with new HCPs.

*“I think there's hesitancy from the young adult to trust their [new] adult provider, because they're worried that they're going to reduce the appointment down to just numbers, as opposed to building the relationship… they're like, ‘can I trust you, are you just going to see my numbers and make a judgment about me as a person based on the fact that my numbers might be slightly elevated?'. And then I think young adults can internalize higher blood sugars or their A1C as a test and whether or not somebody is going to like [them] or not.” [Pediatric HCP]*

*“I'd say maybe like a quarter to a third of them would contemplate canceling or rescheduling an appointment because they don't like where their numbers are.” [Adult HCP]*

*“It can be a bit stressful to see the doctor sometimes.” [Post-Transition AYA, translated]*

HCPs and AYAs described an opportunity to better support the overall physical, mental, and emotional wellbeing of AYAs through more holistic models of transition care by contextualizing more traditional metrics of blood glucose.

*“Like, [in pediatric care] we may be rejoicing in a hemoglobin A1C of 10 where an adult clinic would go “that's terrible, you should be 6”. OK. Well, this kid has been in the 12s or 14s for a couple years.” [Pediatric HCP]*

*“So there's a lot of pressure [to control blood glucose] and I know it made me cry a lot, I hated when my blood sugars were out of control… it's important to kind of tell them “you need to pay attention to this” but maybe try to pressure in a different way.” [Pre-Transition AYA]*

HCPs and AYAs defined a holistic approach to transition care as considering factors beyond diabetes metrics. In this model, an effort is made to get to know the patient, the context around how they live to understand their diabetes management, and the progress made over time to contextualize trends and support them in maintaining good health.

*“I think just generally a more holistic approach to my care would be good. Like looking at me as a person, not just a data point if that makes sense? ” [Post-Transition AYA]*

AYAs view holistic approaches as a facilitator to ease stress and judgment often experienced in clinical settings, and to be more supportive of patient wellbeing and mental health while maximizing AYAs ability to support their diabetes self-management.

*“Diabetes is really about the reality you live with every day. It's not only about seeing the blood sugar on the chart. All those numbers can be explained [by] what you do in your everyday life… being able to have an overall portrait [of] the situation [is] very important.” [Post-Transition AYA]*

HCPs also note how holistic approaches facilitate AYA's ability to support self-care through reducing stress, increasing comfort of AYA with their HCPs, and encouraging appointment attendance by removing the fear of judgment.

*“My initial approach with these patients – it's more let's get to know each other. I'm not going to pass any sort of judgment … And I don't want them to feel that the expectation is super high. Because often that may impact their ability to adhere to appointments, or adhere to recommended changes to regiments, things like that.”[Adult HCP]*

*“We need to be able to meet them [where they're at]. Give them the information we can at the time and accommodate them how we can.”* [*Adult HCP*]

## 4. Conclusion

The aim of our study is to explore perspectives of AYAs living with T1D and T1D HCPs about the strengths, challenges, and opportunities of the transition process. We found a lack of standardized approaches in transition care. Clinicians are limited in their ability to provide comprehensive transition education to support AYA in engaging in T1D self-management. While the American Diabetes Association published recommendations in 2011 for the transition from pediatric to adult diabetes care, our results highlight the majority of clinicians interviewed are unaware of these guidelines (Table 1 provides a brief overview). Advancements in transition guidelines, such as the 2022 ISPAD Clinical Practice Consensus Guidelines [29], that outline the potential role of digital health technologies in T1D transition enhance relevance in the hybrid healthcare landscape of today. Nonetheless, our results support acknowledging two barriers to adoption: a lack of awareness of these tools paired with a resource-constrained environment (time and human resource), which further impedes the capacity for uptake.

Our study also sought to understand how digital health can support transition. The dichotomized notion that virtual care can support building relationships or hinder it is not new. Our findings are corroborated through existing work highlighting virtual care as a facilitator to patient-centred care [17] and relationships [32] while removing barriers of availability, access, and affordability [17]. The ISPAD guidelines describe the potential helpful role of digital tools in transition care [16], and newer technologies, when used successfully, have been shown to improve diabetes self-care [33]. However, in line with our results naming concerns around privacy in the use of virtual care, ISPAD also notes potential breach of confidentiality as a limitation for digital tools [16]. Other groups have also reported how virtual diabetes appointments can create a greater sense of disconnect from HCPs [15] and how integrating practices such as allotting extra time to support rapport building is needed [15]. These polarized results provide an opportunity for hybrid care practices to build and strengthen personal connections, while enhancing access and standardized transition care through digital health tools.

Existing literature supports our results which describe both feelings of judgment in clinical environments and opportunities for enhancement through holistic care. Specifically, holistic care supports quality of life and is protective against diabetes distress when it (1) integrates emotional and social supports, (2) includes discussions of work-and education-related issues and personal challenges in clinical practice, and (3) looks beyond the “numbers” [34, 35]. Failure to integrate these elements of holistic care may contribute to poorer outcomes such as suboptimal glycemic management during transition and diabetes distress [34]. One such element is a need for greater awareness among adult HCPs regarding trends in HbA1c among transition-aged AYA. Adult providers see the majority of patients over 30 years of age. However, at transition, HbA1c values are known to be higher among AYA aged 13–17 years (at 8%–9% or more) and recommended target goals are not reached until closer to age 30. This dissonance between expected target goals can cause distress for the adult provider as well [36]. Overall, our results support the notion that diabetes management is significantly impacted by the stressors outside of T1D that AYAs experience in their daily life [35]. There is therefore a need for transition care to consider factors beyond the traditional diabetes metric to improve diabetes-related health outcomes and better prepare AYAs for transition. A recent trial by Caccalvale et al. [19] focused on coordinating transition preparation between parents, HCPs, and AYAs yielded preliminary evidence to support feasibility, acceptability, and preliminary efficacy of a multisystem, hybrid intervention for AYAs with T1D. Similarly, our results highlight how holistic digital health tools may improve outcomes, such as transition preparedness, that are important to AYA through the use of more convenient communication streams (e.g., virtual appointments and text message).

## 5. Opportunities to Support Type 1 Diabetes Transition Care

Using digital tools to deliver transition education and offering the option of hybrid visits may help overcome barriers to patients attending visits and make visits more efficient. In line with Leung et al. [37], participants shared how digital tools and a digitally mediated hybrid approach may help in (1) serving as the mechanism for delivering standardized transition education curriculum in a personalized way, (2) improving ease of implementing standardized transition guidelines (e.g., checklists), (3) supporting dissemination of educational resources for HCPs and AYAs and track progress through curricula, and (4) supporting streamlined messaging capabilities to close communication gaps between AYAs and HCPs. However, the role of digitally mediated hybrid care as well as mechanisms for standardized implementation of hybrid transition care practices across clinics requires further study to avoid AYAs being lost through the transition [12, 38].

While not specific to hybrid care, Iyengar et al. distilled five recommendations for supporting AYAs through transition within adult care settings [2]: coordinating care across pediatric and adult settings; assessing AYAs' knowledge and skills for adult providers to “capitalize on strengths” and intervention needs; emphasizing relationship building alongside glycemic targets; identification of psychosocial needs and strategies to address them; and adopting team-based approaches to support AYAs engagement. This study supports these broad recommendations (opportunities summarized in Table 2). Pilot adoption of our identified opportunities will aid in identifying most effective elements of transition interventions to improve diabetes outcomes.

## Figures and Tables

**Table 1:** Summary of available resources and guidelines for transition care.

Resource (specificity)	Publishing body (year)	Main resource components
ISPAD Clinical Practice Consensus Guidelines: Transition to Adult Care (7.10) (Type 1 diabetes)	International Society for Pediatric and Adolescent Diabetes [29] (2022)	Supportive models of transition include: (1) Structured transition programs (2) Transition coordinators or “patient navigators” (3) Provider continuity between pediatric and adult health care systems (4) Innovative uses of technology (telemedicine, peer support, web-based, and text messaging interventions) (5) Adult receivership practices

Transition Toolkit for professionals and patients (Type 1 diabetes)	Pediatric Endocrine Society [30] (2020)	(1) Checklist of patient counseling activities to initiate across timelines of early-mid, and late-stage transition (2) Personal Health Diary for AYAs to keep track of health records (3) Transition Passport to facilitate transfer of health information to adult care providers

Six core elements of health care transition (Generic)	Got Transition [10] (2020)	(1) Develop a transition and care policy/guide (2) Track and monitor receipt of six core elements (3) Conduct education and assess transition readiness (4) Transition planning (5) Coordinate transfer of care (6) Completion of transfer

Transitions of care: managing pediatric to adult transitions of care (Type 1 diabetes)	Endocrine Society [31] (2019)	(1) Checklist for provider assessment of patient skill set (2) Patient self-assessment of worries, concerns, and burdens (3) Recommendations for how to begin pediatric transition (4) Clinical summary template to facilitate transfer of health information to adult care providers (5) Recommendations for receiving new patients in adult care (6) Guide for developing resources for new patients (7) Information form for AYAs to indicate visitor preferences to adult HCPs

**Table 2:** Opportunities to support transition and diabetes outcomes.

Opportunities	Mitigation strategies	Anticipated downstream impact
Clinics may lack resources to provide comprehensive, standardized transition care	(1) Considerations for local adaptations of existing guidelines to ensure local community needs are met (2) Increased investment in resources to support transition care (3) Build and integrate digital tools to support AYA transition education coordination of care	Transition Readiness Measure: READDY tool [39] Diabetes knowledge Measure: READDY tool—diabetes knowledge subscale [39] Health system navigation Measure: READDY tool—health system navigation subscale [39]

Building strong relationships through virtual care can be challenging, but virtual care can improve access to care	(1) Adopt hybrid models of care to remove barriers of access, availability, and affordability (2) Implement strategies to enhance accessibility of virtual care for individuals with limited access to technologies	Transition readiness Measure: READDY tool [39]

A high emphasis on traditional diabetes metrics can negatively influence attitudes and perceptions of AYA towards diabetes self-care	Adopt holistic care models that consider the physical, mental, and emotional wellbeing of AYA to support self efficacy in diabetes management	Diabetes self-efficacy Measure: SEDM [40] Diabetes distress Measure: diabetes distress scale [41] Diabetes stigma Measure: barriers to diabetes adherence—stigma subscale [42]

## Data Availability

The qualitative and quantitative data used to support the findings of this study are included within the article and the supplementary information files.

## Additional Points

*Limitations*. Our research adds to existing literature on strengths and challenges of T1D transition care, and opportunities to optimize a hybrid visit model and to integrate digital tools in the delivery of transition care. While participants brought a range of lived experiences of AYAs and HCPs across two provinces, our AYA sample primarily represents only a specific subgroup within the general T1D population (AYA sample: 86% White, 68%–77% financially privileged) and additional perspectives of individuals marginalized by race or ethnicity, or those of lower socioeconomic status need to be further determined. More generally, the impact of socioeconomic status remains an opportunity area for more explicit study as the economic status of those who use a subcutaneous insulin pump may not be a proxy for financial privilege in Quebec since insulin pumps are covered by provincial health care for all, if initiated by 18 years of age [43]. Additionally, our sample's AYAs have universal coverage for physician and hospital services which may yield different outcomes than those in other healthcare systems, or where transfer of care occurs at times different from 18 years of age. Similarly, our research experienced a sampling bias of AYAs motivated to contribute to research. While we were unable to capture AYAs who may be considered more “at risk” for experiencing gaps in care during the transition process, we aimed to fill this gap by hearing from HCPs who work with a range of AYAs. *Summary*. Our results highlight three key findings. First, digitally mediated care can enable standardized practices and training to strengthen the knowledge and skills of AYA across transition, and provide parallel integration of assessment tools to identify needs and areas for support. Participants saw value in the opportunity to leverage digital tools to facilitate the implementation of transition guidelines and enhance team-based transition care. Second, provision of hybrid transition care can improve accessibility to pediatric and adult care. However, consideration should be given to reducing barriers to virtual care implementation and participation. Third, AYAs and HCPs emphasized the importance of holistic care and the need to respond to HbA1c and other measures of glycemia while taking into consideration the context of AYAs lives beyond T1D. Integrating physical, mental, and emotional wellbeing across transition care is a key to building and strengthening relationships between AYAs and their HCPs while supporting AYA self-efficacy and capacity for diabetes self care.

## Supplementary Materials

Supplementary 1 File 1: team positionality.

Supplementary 2 File 2: interview guides.

Supplementary 3 File 3: demographic questionnaires.

Supplementary 4 Table 1: demographic information and diabetes experiences for participants living with Type 1 diabetes.

Supplementary 5 Table 2: demographic information and diabetes experience for healthcare provider participants.
