# Supplementary material for: Supporting Adolescents and Young Adults through Digitally Mediated Type 1 Diabetes Transition Care: A Qualitative Descriptive Study
Source: Pediatr Diabetes. 2024 Jul 15;2024:3721768. doi: 10.1155/2024/3721768 (PMC12017227; doi:10.1155/2024/3721768)
Supplement: Supplementary 1 — File 1: team positionality. [file 3721768.f1.docx]

# **Supplemental File F1:** Team Positionality

Author NE was born, raised, and lives and works in the Greater Toronto Area, where her parents immigrated to from Lebanon. Conducting this research, her position as a young professional helped her better connect with and relate to the YA participants, which she felt made interviews more personal and comfortable. As someone who does not live with Type 1 Diabetes and had limited knowledge of Type 1 Diabetes before beginning this project, she recognizes the limitation of her perspectives and worked hard to listen to, learn from, and centre the experiences of those who lived with Type 1 Diabetes in the analysis and presentation of their stories.

Author MT is a settler on Turtle Island of European descent, born, raised and residing on the lands now referred to as the Greater Toronto Area. She and her family have had many interactions with the Ontario healthcare system in both the pediatric and adult settings which she feels guided her empathy when conducting interviews. Being that she is a young professional, she feels that her ability to relate to the YA fostered a genuine environment of learning where she sought to authentically listen and understand the experiences shared by all participants. To our participants: please know your stories are held in the utmost care.

Author AS supported the team with analysis of interviews, and he acknowledges that that way that he analyzed the interviews is shaped by his positionality. His perspective is shaped by the fact that he is a visible minority who was born in a rural village in India and grew up in India, USA, and Canada. In addition, he is a male who is currently pursuing a bachelor’s degree in Health Science and part of the lower middle class.
