# Supplementary material for: Supporting Adolescents and Young Adults through Digitally Mediated Type 1 Diabetes Transition Care: A Qualitative Descriptive Study
Source: Pediatr Diabetes. 2024 Jul 15;2024:3721768. doi: 10.1155/2024/3721768 (PMC12017227; doi:10.1155/2024/3721768)
Supplement: Supplementary 2 — File 2: interview guides. [file 3721768.f2.docx]

# Supplemental File F2: Interview Guides

Interview Questions for Pre-Transition YA

| **Learning to Become Independent** | This first set of questions is about understanding what type of support you’re getting to help manage diabetes. This support can come from family, friends, or healthcare providers like doctors, nurses, social workers, and dieticians.   1. Please tell me about your experience managing your diabetes over the past few years and more recently as you prepare to transition to adult care. 2. Do you have the confidence to manage your diabetes by yourself? 3. What aspects of your diabetes do you currently manage on your own and what do you generally get help for (e.g., insulin injections, boluses and adjustments)? 4. The people who help you with diabetes management, whether they are family, friends, or providers, are referred to as a “support network”. Can you tell me about your different support networks and how they help you with your diabetes care? 5. How and to whom do you voice diabetes-care related concerns to? 6. In what ways do you feel supported in your diabetes management by your healthcare providers? 7. In what ways do you feel supported in your diabetes management by your family and friends? 8. Which support networks do you value the most?    1. Family, friends, healthcare providers, or all equality? 9. To what degree do you want your parents, family, or friends involved in your diabetes care? 10. Are there certain things you don’t want them to be involved in? 11. In what ways do you think your support networks will be different during or after your transition to adult care? 12. Do you think the support you get from your healthcare providers will be different during or after your transition to adult care? 13. Do you think you will be seen at more than one adult site (eg., school, home town)? 14. Do you think there will be a change in the way your family and friends support you in your diabetes management when you start being seen in an adult setting? 15. How do you intend to go about rebuilding support networks in an adult care setting? 16. Do you feel that you have all the information you need to build a good support network? If not, what information are you missing? 17. How do you feel about attending your first adult appointment? 18. Do you feel confident that you will be able to attend your first scheduled appointment? 19. Why or why not? 20. Is there anything that would make you feel more confident? 21. How do you feel about managing your diabetes independently? |
| --- | --- |
| **Compilation of Stressors-** | These next questions are about trying to understand life challenges that might be directly caused by diabetes or that make your diabetes management more difficult.   1. What are some things in your life that cause you stress? 2. Does anything about your diabetes management cause you stress? 3. What about managing your diet and blood sugars? 4. What about using diabetes technology (pumps, CGMs, flash monitors)? 5. Are there any challenges in other aspects of your life (e.g. school, work, relationship) that cause you stress? 6. Do you have any resources or supports to help you deal with things that cause you stress? 7. What are you most worried about when it comes to transitioning to adult care? 8. What do you think it will be like to receive care in an adult setting? 9. Do you worry about needing to become more independent in the management of your T1D? 10. Do you have any unanswered questions about how things will work once you start going to your adult clinic? 11. Do you have any worries about getting funding or insurance coverage for insulin or diabetes supplies and technology (pumps, CGMs, flash monitors)? 12. Do any aspects of the upcoming transition process create additional stress in your life? 13. Mental health support resources can help with stress management, do you know what types of mental health supports are available to you if you need it? 14. Have you made use of these supports? If so, how helpful were they, why or why not? 15. How did you become aware of these resources? Did someone tell you about them or did you find them through your own research? |
| **Establishing relationships with adult care (Trust, Communication)** | During your transition process, the members of your diabetes healthcare team will likely change. The next questions are about the relationships between you and your current healthcare team and what you expect the relationships will be like with your adult care provider.   1. How would you describe your relationship with your current diabetes healthcare provider team? 2. Do you feel that your provider team listens to your experiences and preferences when they make recommendations about your diabetes care? 3. Is there anything you are not comfortable discussing with your diabetes providers? 4. If so, what might improve your relationship or level of comfort with your current provider team? 5. In what ways do you expect your relationships with your adult providers to be different from the relationships you have with your current providers? 6. Can you think of anything that could help build strong relationships with your future adult provider? 7. Do you want your adult providers to have information about you and your diabetes history or would you rather have a fresh start? 8. What is the most important information that you want them to know? 9. Are there any topics you won’t feel comfortable discussing with your adult providers until you build a relationship with them? 10. Do you think you are getting the best care possible or can you think of ways your diabetes care could be improved? 11. Do you think there is anything missing, or gaps, in the care you receive at your current diabetes care setting? If so, what are they? 12. Do you think these gaps in care will change during your transition to an adult care setting? How so? 13. Do you think there will be new gaps in care after your transition to adult care?  If so, what are they? |
| **How can we create a robust and cost-effective intervention?** | The last set of questions will explore the potential of a new smartphone tool to support patients in their transition to adult care.   1. What form of digital communication do you prefer? 2. Text messaging, email, phone call, social media (e.g., Facebook or Instagram)? 3. Do you have a mobile phone or device that is able to receive text messages? 4. What happens if you are travelling or do not have connectivity – how do you prefer to be contacted then? 5. If you were to receive text messages to your mobile phone with information aimed at supporting your transition to adult care, what type of support and content would you be (/have been) interested in receiving? 6. When or how often you like to receive this information? 7. Are there other people in your life you would want to receive (/have received) that same information? 8. Would you want to be responsible for sharing that information with them or would you want them to receive it at the same time as you? |

**Interview Questions for Post-Transition YA**

| **Learning to Become Independent – Life Changes, Readiness** | This first set of questions is about understanding what type of support you’re getting to help manage diabetes. This support can come from family, friends, or healthcare providers like doctors, nurses, social workers, and dieticians.   1. Please tell me about your experience transitioning from pediatric to adult care. 2. Where are you currently being followed for your T1D? 3. Were you originally referred to another adult care site before you started being seen at _____ clinic? If so, how did you end up being followed here instead? 4. The people who help you with diabetes management, whether they are family, friends, or providers, are referred to as a “support network”. Can you tell me about your different support networks and how they help you with your diabetes care? 5. How and to whom do you voice diabetes-care related concerns to? 6. In what ways do you feel supported in your diabetes management by your healthcare providers? 7. In what ways do you feel supported in your diabetes management by your family and friends? 8. Which support networks do you value the most? 9. Family, friends, healthcare providers, or all equality? 10. To what degree do you want your parents, family, or friends involved in your diabetes care? 11. Are there certain things you don’t want them to be involved in? 12. Did your support networks change during or after the transition to adult care? 13. In what ways did the support you get from your healthcare provider team change? 14. In what ways did the support you get from your family and friends change? 15. In what ways has your access to resources changed since transitioning to adult care (e.g., people, health insurance, healthcare services, etc.) 16. Do you or did you feel the need to rebuild support networks after your transition to adult care? 17. How do you intend to go about rebuilding these support networks? (if they already did: What did you do rebuild these support networks?) 18. Do you feel that you have all the information you need to rebuild these support networks? 19. If not, what information or resources are you missing? 20. How many months had gone by between your last pediatric appointment and your first adult appointment? 21. Did you communicate with anyone from your old pediatric care team during that time? 22. Did you communicate with anyone in your new adult care team during that time? 23. How long have you been in adult care? 24. How independent do you feel when it comes to managing your own diabetes care? 25. Do you feel dependent on your support networks that we discussed earlier or do you feel confident in being the main person in charge of your diabetes? 26. How has your level of independence changed since your first visit with your adult team? Why do you think that is? |
| --- | --- |
| **Compilation of Stressors** | These next questions are about trying to understand life challenges that might be directly caused by diabetes or that make your diabetes management more difficult.   1. What are some things in your life that cause you stress? 2. Does anything about your diabetes management cause you stress? 3. What about managing your diet and blood sugars? 4. What about using diabetes technology (pumps, CGMs, flash monitors)? 5. Are there any challenges in other aspects of your life (e.g. school, work, relationship) that cause you stress? 6. Do you have any supports (e.g., people, information, health services) to help you deal with things that cause you stress? 7. Please tell me about any challenges you faced in your transition to adult care. 8. Have you undergone any changes in your life that had an impact on your day-to-day diabetes management? How so? 9. Have you undergone any changes in your life that made it difficult for you to transition from pediatric to adult care? 10. Did you face any challenges getting funding or insurance coverage for insulin or diabetes supplies, or technology (pumps, CGMs, flash monitors)? 11. Did any aspects of the transition process (either before, during, or after) create additional stress in your life? 12. Prior to your first adult clinic visit, what were you expecting would happen? 13. How was your first adult clinic visit? 14. In what ways was it different from what you expected? 15. What did you like about it? 16. What did you dislike about it? 17. Did you feel prepared? 18. Mental health support resources can help with stress management, do you know what types of mental health supports are available to you if you need it? 19. Have you made use of these supports? If so, how helpful were they, why or why not? 20. How did you become aware of these resources? Did someone tell you about them or did you find them through your own research? |
| **Establishing relationships with adult care (Trust, Communication)** | During your transition to adult care, you likely had to change your diabetes healthcare team. The next set of questions are focused on your relationship with your former pediatric providers and your new adult healthcare teams.   1. How would describe your relationship with your previous pediatric diabetes providers? 2. Did you feel that your provider team listened to your experiences and preferences when they made recommendations about your diabetes care? 3. Is there anything you were not comfortable discussing with them? 4. If so, what might have improved your relationship or level of comfort with your current provider team? 5. Please describe your relationship with your current adult providers 6. Do you feel that your adult providers listen to your experiences and preferences when they make recommendations about your diabetes care? 7. If you have more than one adult provider, how would you describe the relationship between your adult providers? 8. Can you describe what the relationship-building process looked like with your adult care providers? 9. What factors were most helpful when building your relationship with the adult provider? 10. Are there any topics you don’t feel comfortable discussing with your adult provider? 11. In your opinion, what type of environment is the most conducive to developing a healthy and effective patient-provider relationship? 12. Do you think you are getting the best care possible or can you think of ways your diabetes care could be improved? 13. Is there anything missing in the care you receive in your adult care setting? 14. Are these gaps in care new since transitioning to adult care or did they exist when you were followed in a pediatric care clinic? |
| **How can we create a robust and cost-effective intervention?** | The last set of questions will explore the potential of a new smartphone tool to support patients in their transition to adult care.   1. What form of digital communication do you prefer? 2. Text messaging, email, phone call, social media (e.g., Facebook or Instagram)? 3. Do you have a mobile phone or device to be able to receive text messages? 4. What happens if you are travelling or do not have connectivity – how do you prefer to be contacted then? 5. If you were to receive text messages to your mobile phone with information aimed at supporting your transition to adult care, what type of support or information would you have been interested in receiving? 6. When or how often would you have liked to receive this information? 7. Are there other people in your life you would have wanted to receive that same information? 8. Would you have wanted to be responsible for sharing that information with them or would you want them to receive it at the same time as you? |

**Interview Questions for Pediatric HCP**

| **Learning to Become Independent – Life Changes, Readiness** | In addition to the transfer of care to an adult provider, many consider transition to include the gradual increase in patient independence when it comes to managing their diabetes. The first set of questions is focused on your experience as a provider during the transition process.   1. How do you define readiness to transition? 2. What is this definition based on (eg. professional guidelines, experience, standardized tool, etc.)? 3. How do you determine if an individual is adequately prepared to transition to adult care? 4. In your opinion, what factors in an individual’s life might hinder readiness to transition? (e.g. competing priorities) 5. What do you, as a pediatric T1D provider, perceive your responsibilities to be in terms of guiding patients toward independence in navigating all aspects of their diabetes management? 6. What goals do you have for your patients as they transition into adult care? 7. Please describe what you and your pediatric centre do to help prepare your patients to transition to adult care? 8. When (how early) does this preparation start? 9. What is the nature of the conversations you have with patients? 10. Do you discuss the nature of adult care? 11. Does the pediatric centre where you work have a standardized protocol for transition care? 12. Does the preparation look different depending if the patient is using a pump or insulin injection? 13. To your knowledge, what are the existing local, provincial, and national resources to help patients transition from pediatric to adult care? 14. How and by whom are patients being made aware of these resources? 15. To your knowledge, what are the existing local, provincial, and national resources to help you with best practices, particularly as it relates to the role of pediatric T1D providers in the transition process? 16. How would you describe the T1D patient population you currently serve (e.g., age, gender, sex, race, household composition, social deprivation index)? 17. Some patients might experience additional burdens to transitions. For example, some might struggle with becoming independent, some might be more prone to anxiety, and some might be more likely to miss clinic appointments. With this in mind, have you noticed that patients with certain demographic or personality characteristics are disproportionately burdened during transition? |
| --- | --- |
| **Compilation of Stressors- support, information overload** | The next questions are about how healthcare providers navigate providing mental health and psychosocial support to those who require it during the transition period.   1. What types of mental health challenges have you witnessed in your patients as they transition to adult care? 2. What symptoms of psychological distress have you noticed when patients think about transitioning? How common are they? 3. What kind of resources are available to you, as a T1D provider, in terms of assessing and addressing these types of psychosocial or mental health issues? 4. Have you ever received training on how to support patients with their psychosocial stressors? 5. At what point, after noticing these psychosocial challenges, do you refer your patients to another medical or mental health provider? |
| **Establishing relationships with adult care (Trust, Communication)** | The following questions are about your relationship with pediatric patients and the challenges they might face in building new relationships with the adult providers.   1. How do you describe your relationship with patients in the year leading up to the transfer to an adult setting? 2. Do you find that this relationship typically changes since you first start following them? If so, why do you think that is? 3. What is your degree of engagement with patients outside of clinic visits? 4. Do you communicate with your patients outside of in-person clinic visits or outside of work hours? 5. In your opinion, what are the foundations or key elements of a good patient-provider relationship? 6. What challenges do you think recently transitioned patients might face in establishing good patient-provider relationships with their new adult care team? 7. Are there certain things that adult providers need to consider when attempting to establish trust and effective communication with recently transitioned young adults? 8. What does your involvement look like between the last pediatric clinic visit and the first adult clinic visit? 9. Do you follow up with your patients in any way? 10. If a patient is transitioning and they contact you regarding a diabetes-related problem, what role do you play? |
| **How can we create a robust and cost-effective intervention?** | The last set of questions will explore the potential of a new smartphone tool to support patients in their transition to adult care.   1. In your opinion, what can be improved to increase the likelihood of successful transitions? 2. What resources or supports do patients need that fall outside the scope of what existing provider teams are expected to deliver? 3. What resources could enhance your ability to do more in the context of your existing role? 4. Based on these discussions, we will be designing a mobile phone intervention to support patients in their transition. Can you think of any features that would be useful to include? 5. If the mobile phone intervention could administer a readiness survey to patients, would you be interested in seeing those results? 6. How can this information be presented to you in a meaningful and useful way? 7. What actions could you take in response to those results (i.e. what would you do if the patient has low readiness)? 8. Key milestones in the transfer to adult care include sending the referral, the final pediatric clinic visit, and the first adult clinic visit. With this in mind, what kind of information would you find useful to receive at the time of, or between these different transition milestones? |

**Interview Questions for Adult HCP**

| **Learning to Become Independent – Life Changes, Readiness** | The first set of questions is focused on your experience as a provider during the transition process.   1. How long after moving out of pediatric care do you believe patients begin to feel independent in their own care? 2. How would you describe the degree of readiness when they first visit your practice? 3. At what point do you consider your patients to be fully independent? 4. What do you, as an adult T1D provider, perceive your responsibilities to be in terms of guiding patients toward independence in navigating all aspects of their diabetes management? 5. What goals do you have for your patients as they transition into adult care? 6. Please describe the first intake appointment with newly transitioning patients? 7. What is the focus of this first visit? 8. Do you do anything or review information to prepare for this first visit? If so, what? 9. What is spoken about? 10. How long does it last? 11. What do you perceive patients’ expectations to be? 12. Are different things covered depending on if the patient is using a pump or insulin injection? 13. To your knowledge, what are the existing local, provincial, and national resources to help patients transition from pediatric to adult care? 14. How and by whom are patients being made aware of these resources? 15. To your knowledge, what are the existing local, provincial, and national resources to help you with best practices, particularly as it relates to the role of adult T1D providers in the transition process? 16. How would you describe the T1D patient population you currently serve (e.g., age, gender, sex, race, household composition, social deprivation index)? 17. Some patients might experience additional burdens to transitions. For example, some might struggle with becoming independent, some might be more prone to anxiety, and some might be more likely to miss clinic appointments. With this in mind, have you noticed that patients with certain demographic or personality characteristics are disproportionately burdened during transition? |
| --- | --- |
| **Compilation of Stressors- support, information overload** | The next questions are about how healthcare providers navigate providing mental health and psychosocial support to those who require it during the transition period.   1. What types of mental health challenges have you witnessed in your patients when they first transition to adult care, if any? 2. What symptoms of psychological distress have you noticed? How common are they? 3. What kind of resources are available to you, as a T1D provider, in terms of assessing and addressing these types of psychosocial or mental health issues? 4. Have you ever received training on how to support patients with their psychosocial stressors? 5. At what point, after noticing these psychosocial challenges, do you refer your patients to another medical or mental health provider? |
| **Establishing relationship with adult care (Trust, Communication)** | Patients typically change diabetes healthcare providers when to transition to adult care, requiring the building of new patient-provider relationships. The following questions are about your relationship with newly transitioning patients?   1. How do you describe your relationship with patients when you first start seeing them in an adult setting? 2. Do you find that this relationship typically changes over time? If so, why do you think that is? 3. What is your degree of engagement with patients outside of clinic visits? 4. Do you communicate with your patients outside of in-person clinic visits or outside of work hours? 5. If a patient experiences a diabetes-related problem between visits, what role do you play? 6. Do you believe your patients’ relationships were different with their pediatric providers? How so? 7. In your opinion, what are the foundations or key elements of a good patient-provider relationship? 8. Do any of your “transitioning” patients get referred to you from other adult clinics instead of coming straight from pediatric care? 9. If so, what are some of the reasons for this? 10. What type of information do you get about the patient from the other adult provider along with the referral? 11. Are any of your patients followed by multiple adult endocrinologists/ providers? 12. What are some examples of what would cause this situation to happen? 13. How do you collaborate with these other endocrinologists? |
| **How can we create a robust and cost-effective intervention?** | The last set of questions will explore the potential of a new smartphone tool to support patients in their transition to adult care.   1. In your opinion, what can be improved to increase the likelihood of successful transitions? 2. What resources or supports do patients need that fall outside the scope of what existing provider teams are expected to deliver? 3. What resources could enhance your ability to do more in the context of your existing role? 4. Based on these discussions, we will be designing a mobile phone intervention to support patients in their transition. Can you think of any features that would be useful to include? 5. What kind of information would you find useful to have as patients transfer into your care? 6. What would you like to know when you receive the referral from the pediatric provider? 7. What would you like to know after a patient’s final pediatric clinic visit? 8. How would you act on these pieces of information? 9. Key milestones in the transfer to adult care include receiving the referral, the final pediatric clinic visit, and the first adult clinic visit. With this in mind, what kind of information would you find useful to receive at the time of, or between these different transition milestones? |
