# Supplementary material for: Supporting Adolescents and Young Adults through Digitally Mediated Type 1 Diabetes Transition Care: A Qualitative Descriptive Study
Source: Pediatr Diabetes. 2024 Jul 15;2024:3721768. doi: 10.1155/2024/3721768 (PMC12017227; doi:10.1155/2024/3721768)
Supplement: Supplementary 3 — File 3: demographic questionnaires. [file 3721768.f3.docx]

# Supplemental File F3: Demographic Questionnaires

**Demographic Questionnaire – Patients**

How old are you? ____

Please select your gender

1. Male
2. Female
3. Pass
4. Other

What is your ethnicity?

1. Indigenous (Inuit, Metis, North American Indian)
2. Arab/West Asian (e.g. Armenian, Egyptian, Iranian, Lebanese, Moroccan)
3. Black (e.g. African, Haitian, Jamaican, Somali)
4. Chinese
5. Filipino
6. Japanese
7. Korean
8. Latin American
9. South Asian
10. South East Asian
11. White (Caucasian)
12. Pass
13. Other

What province do you currently live in?

1. Quebec
2. Ontario
3. Pass

What type of area do you live in?

1. Urban (in a city)
2. Suburban (in a community adjacent to a city)
3. Rural (in a small town)
4. Pass

How long have you been living with diabetes?

1. 0-2 years
2. 3-5 years
3. 6-10 years
4. 11-15 years
5. 16-20 years
6. 20+ years
7. Pass

How would you rate your blood glucose management?

1. Excellent
2. Good
3. Neutral
4. Fair
5. Poor
6. Pass

What diabetes devices do you use? (you can choose more than one)

1. Insulin pump
2. Syringe
3. Pen
4. Continuous glucose monitor (CGM)
5. Glucometer
6. Pass

What insulin pump brand do you use? _______

What continuous glucose monitor (CGM) brand do you use? ________

What glucometer brand do you use? _______

How long does it take to travel to your pediatric endocrinologist?

1. 0-15 minutes
2. 15-30 minutes
3. 30-45 minutes
4. 60-90 minutes
5. +90 minutes
6. Pass

How would you rate your relationship with your pediatric endocrinologist?

1. Excellent
2. Good
3. Neutral
4. Fair
5. Poor
6. Pass

Select all that apply, who you do see regarding your diabetes management?

1. Endocrinologist
2. Nurse
3. Dietician
4. Social Worker
5. Psychologist
6. Pass
7. Other

What is your biggest struggle(s) living with T1D? (Select all that apply)

1. Affording insulin supplies
2. Communication with health care providers
3. Difficulty connecting with other T1D’s
4. Lack of resources (education, information, etc…)
5. Managing blood glucose
6. T1D stigma
7. Self-management
8. Relationship with my parents
9. Making new friends
10. Managing school work
11. Work-life balance
12. Pass
13. Other

If you plan to transition soon, or are in the middle of transitioning, how do you feel about the transitioning from pediatric to adult clinical care? If you have already transitioned, please select “I have already transitioned”.

1. Confident, not worried
2. I sometimes worry
3. Neutral, never thought of it
4. I am worried
5. Stressed, I’m extremely worried
6. I have already transitioned
7. Pass
8. Other

If you have already transitioned, how do you feel about your transition from pediatric to adult clinical care? If you have not yet transitioned, please select “I have not transitioned”.

1. Excellent, it was the best it could have been
2. Good, there were a few areas of improvement
3. Neutral, it was fine
4. Not good, there were several areas of improvement
5. Very poor, you did not feel supported
6. Pass
7. I have not yet transitioned
8. Other

**Demographic Questionnaire – Healthcare providers**

Please select your gender

1. Male
2. Female
3. Pass
4. Other

Do you practice in Ontario or Quebec?

1. Ontario
2. Quebec
3. Pass

Describe the geographic region in which you practice.

1. Urban (in a city)
2. Suburban (in a community adjacent to a city)
3. Rural (in a small town)
4. Pass

What role do you play in the clinical care team?

1. Nurse
2. Dietician
3. Endocrinologist
4. Social worker
5. Pharmacist
6. Administrator
7. Pass
8. Other

What percentage of your patients have T1D (vs T2D)?

1. 100-90%
2. 89-75%
3. 74-50%
4. 49-25%
5. 24-10%
6. <10%
7. None of my patients
8. Pass

What is the age range of most of your T1D patients (check all that apply)

1. <18 years old
2. 18-25 years old
3. 25-35 years old
4. 35-55 years old
5. 55 years +
6. Pass

How long have you been working with patients who have T1D?

1. 1-5 years
2. 6-10 years
3. 11-15 years
4. 16-20 years
5. 20+ years
6. Not applicable
7. Pass

How would you rate the current transition process from pediatric to adult care for people living with T1D?

1. Excellent
2. Good
3. Neutral
4. Fair
5. Poor
6. Not applicable
7. Pass

What platforms do you use to communicate with your patients?

1. Telephone
2. Email
3. Text message (SMS)
4. Mail (i.e. Canada post, UPS)
5. Fax
6. None of the above, I only communicate with patients in person at our clinic
7. Pass
8. Other
