# Supplementary material for: Supporting Adolescents and Young Adults through Digitally Mediated Type 1 Diabetes Transition Care: A Qualitative Descriptive Study
Source: Pediatr Diabetes. 2024 Jul 15;2024:3721768. doi: 10.1155/2024/3721768 (PMC12017227; doi:10.1155/2024/3721768)
Supplement: Supplementary 4 — Table 1: demographic information and diabetes experiences for participants living with Type 1 diabetes. [file 3721768.f4.docx]

# Supplemental Table S1

**Table S1:** Demographic information and diabetes experiences for participants living with Type 1 Diabetes (n=22)

| **Variable** | | **N** | **%** |
| --- | --- | --- | --- |
| **Age (years)** | 16-17 | 11 | 50 |
|  | 18-25 | 11 | 50 |
| **Gender** | Female | 14 | 64 |
|  | Male | 8 | 36 |
| **Ethnicity** | White (Caucasian) | 19 | 86 |
|  | Other | 3 | 14 |
| **Province of residence** | Ontario | 11 | 50 |
|  | Quebec | 11 | 50 |
| **Years living with Type 1 Diabetes** | 0-5 years | 5 | 23 |
|  | 6-15 years | 12 | 55 |
|  | 16+ years | 5 | 23 |
| **Use of insulin delivery technology*** | Insulin pump | 17 | 77 |
|  | Insulin injections | 7 | 32 |
| **Use of glucose monitoring technology*** | Continuous Glucose Monitor (CGM) | 15 | 68 |
|  | Glucometer | 8 | 36 |
| **Travel time to YA’s endocrinologist (minutes)**** | 0-15 | 3 | 14 |
|  | 15-30 | 8 | 36 |
|  | 30-45 | 6 | 27 |
|  | 60-90 | 3 | 14 |
| **Biggest challenges faced in living with Type 1 Diabetes***** | Communication with healthcare providers | 5 | 23 |
|  | Difficulty connecting with others living with Type 1 Diabetes | 6 | 27 |
|  | Managing blood glucose | 14 | 64 |
|  | Facing stigma associated with Type 1 Diabetes | 6 | 27 |
|  | Self-management of Type 1 Diabetes | 11 | 50 |
|  | Managing school work | 5 | 23 |
|  | Work-life balance | 5 | 23 |

*Proxy for proportion being financially privileged since insulin pumps and CGM incur an additional expense

**Indicates missing response(s)

**Participants could select multiple options
