# Supplementary material for: Supporting Adolescents and Young Adults through Digitally Mediated Type 1 Diabetes Transition Care: A Qualitative Descriptive Study
Source: Pediatr Diabetes. 2024 Jul 15;2024:3721768. doi: 10.1155/2024/3721768 (PMC12017227; doi:10.1155/2024/3721768)
Supplement: Supplementary 5 — Table 2: demographic information and diabetes experience for healthcare provider participants. [file 3721768.f5.docx]

# Supplemental Table S2

**Table S2:** Demographic information and diabetes experience for healthcare provider participants (n=21)

| **Variable** | | **N** | **%** |
| --- | --- | --- | --- |
| **Province of practice** | Ontario | 16 | 76 |
|  | Quebec | 5 | 24 |
| **Clinical role** | Endocrinologist | 5 | 24 |
|  | Nurse | 5 | 24 |
|  | Dietitian | 5 | 24 |
|  | Other (Social worker, pharmacist, general pediatrician) | 6 | 29 |
| **Proportion of patients in your practice with Type 1 Diabetes (%)** | < 25% | 5 | 24 |
|  | 25-74% | 2 | 10 |
|  | >=75% | 14 | 67 |
| **Ages of patients in your practice (years)** | Under 18 | 14 | 67 |
|  | 18 - 25 | 8 | 38 |
|  | 25 years or older | 16 | 76 |
| **Experience providing Type 1 Diabetes care (years)** | 1-5 | 7 | 33 |
|  | 6-10 | 6 | 29 |
|  | 11+ | 8 | 38 |
